# A Novel Dual-Functional Photomultiplication-Type Organic Photodetector with Photon-Regeneration Gain by Integrated Organic Light-Emitting Diodes

**DOI:** 10.34133/research.1094

**Published:** 2026-01-26

**Authors:** Ji Li, Liqing Yang, Guo He, Jinghao Fu, Rentao Dong, Dechao Guo, Dezhi Yang, Dongge Ma

**Affiliations:** Institute of Polymer Optoelectronic Materials and Devices, Guangdong Basic Research Center of Excellence for Energy & Information Polymer Materials, Guangdong-Hong Kong-Macao Joint Laboratory of Optoelectronic and Magnetic Functional Materials, State Key Laboratory of Luminescent Materials and Devices, South China University of Technology, Guangzhou 510640, China.

## Abstract

Because of their outstanding weak light detection ability, multiplication-type photodetectors have great application prospects in fields such as environmental detection, biological science, and night vision imaging. Traditional photomultiplication-type organic photodetectors (PM-OPDs) usually achieve a large external quantum efficiency (*EQE*) through the mechanism of interfacial trap-assisted charge tunneling injection but inevitably produce a large dark current. Here, we demonstrate a novel dual-function photon-regeneration multiplication-type organic photodetector (PRM-OPD) with photocurrent gain by absorbing additional photons generated by an integrated organic light-emitting diode unit. The optimized PRM-OPD exhibits a maximum *EQE* of 2,484% and maintains a low dark current density (*J_d_*) of only 10^−8^ A/cm^2^. More importantly, the resulting PRM-OPD can not only exhibit efficient detection performance but also directly detect the shape of the light spot, showing a dual-function working mode. Furthermore, by combining optical resonant microcavities, the response spectrum is successfully extended to the near-infrared region and a narrowband PRM-OPD with excellent performance is obtained. This designed device structure provides a new idea for the development of high-performance PM-OPDs.

## Introduction

In recent years, there has been a significant interest in organic photodetectors (OPDs) due to their potential applications, including image sensing, health monitoring, and optical communication [1–8]. Compared with conventional inorganic photodetectors, OPDs offer several advantages such as adjustable optical bandgaps, mechanical flexibility, solution processability, and low production costs. Moreover, previous studies have demonstrated various dual-functional operation modes in OPDs, including voltage-polarity-tunable spectral response ranges (e.g., broadband/narrowband switching via forward/reverse bias) [9–11] and bias-amplitude-modulated operation modes (e.g., photovoltaic vs. photomultiplication [PM] modes) [12–14]. These dual-functional designs not only expand the application scope of organic optoelectronic devices but also enhance environmental adaptability through dynamic mode switching.

As the photoelectric detection systems advance toward high definition, high resolution, and miniaturization, the number of photons received by a single photosensitive pixel has decreased, leading to reduced sensitivity to weak light signals. OPDs currently face the challenge of insufficient sensitivity in detecting weak light signals, which is similar to their inorganic counterparts. To tackle this issue, it is crucial to enhance quantum efficiency and reduce the dark current, thereby improving overall sensitivity. In general, a conventional inorganic silicon photodetector can generate a maximum of one electron–hole pair upon photon absorption, resulting in an external quantum efficiency (*EQE*) that does not exceed 100%. However, the constraint is overcome by the avalanche effect. By operating under a high reverse bias, the photogenerated carriers are accelerated by the electric field and undergo ionization through collisions, thus generating additional charge carriers and achieving a substantial photocurrent gain with high sensitivity. Unfortunately, this mechanism is not applicable in organic semiconductors due to their inherent characteristics, such as low dielectric constant, high exciton binding energy, and internal structural disorder. Nevertheless, the photocurrent gain in OPDs was still observed in organic pigmented films sandwiched between Au electrodes [15]. This phenomenon is attributed to interfacial trap-assisted charge tunneling injection, which has become the predominant strategy for achieving PM in OPDs. Based on this mechanism, a variety of photomultiplication-type organic photodetectors (PM-OPDs) have been developed. The typical strategies for achieving photocurrent gain in these PM-OPDs generally involve introducing trap states into the active layer or interface layer between the electrodes and organic materials and utilizing an interface barrier layer to hinder the collection of electrons or holes [16–20]. However, achieving a low dark current density (*J_d_*) as well as high photocurrent gain is challenging for such devices, and their high detection capability typically depends on a high *EQE* [21–34]. We have summarized representative PM-OPDs with an *EQE* exceeding 1,000% and a *J_d_* below 10^−5^ A cm^−2^ over the past 5 years, as shown in Table S1 (Supplementary Materials). Therefore, it is imperative to explore novel PM mechanisms and device structures that can effectively suppress dark currents, thus ultimately obtaining high-performance PM-OPDs.

In our previous work, we pioneered the concept of using an OPD unit and a tandem organic light-emitting diode (OLED) unit to fabricate organic up-conversion devices, which convert invisible near-infrared (NIR) light to visible light, and demonstrated that visible light reabsorption markedly enhances their performance. This led to integrated devices achieving a peak photon-to-photon conversion efficiency of 29.6% [35]. It was observed that the photoactive layer in the OPD unit can absorb photons emitted by the OLED unit and convert them into charge carriers, which can be understood as optical feedback. This mechanism leads to an *EQE* of the up-conversion device that exceeds that of the OPD alone. Although the above up-conversion device has achieved an improvement in light-to-light conversion efficiency, its photoelectric conversion efficiency has not exceeded 100%, thereby failing to realize multiplicative photodetection gain. Building upon prior work, we in this work employ the photon-regeneration principle to realize gain devices featuring a low dark current density. Importantly, compared with the traditional gain mechanism [36,37], the gain mechanism here does not rely on the intentional introduction of trap states; the dark current remains governed by the OPD unit, which is similar to conventional photodiodes, thus enabling the realization of photodetectors with both internal gain and a low dark current. Meanwhile, such devices do not require the construction of defects to achieve gain, which undoubtedly reduces the difficulty of structural design and fabrication. Due to the presence of an optical feedback mechanism, this gain-based device will also exhibit a dual functionality for detecting light spots. Currently, the characterization of light spot morphology and intensity distribution still relies on charge-coupled devices/complementary metal–oxide–semiconductor sensors or beam profilers, which increases the complexity of the system. Therefore, integrating photocurrent gain and the ability to directly capture the shape of the light spot within a single OPD could simplify the complex detection system while simultaneously achieving high signal-to-noise ratio measurement.

In this study, we fabricated photon-regeneration multiplication-type OPDs (PRM-OPDs) by integrating an efficient OLED unit with a conventional OPD unit. It is found that the photogenerated carriers from the OPD unit are transported to the OLED unit to generate additional photons, which are then subsequently absorbed by the OPD unit to enhance the photocurrent response. Because this process can be repeated during illumination and applied bias, as a result, a continuous increase in the number of photons generated within the OLED unit is achieved, thus leading to a substantial gain in photocurrent. The optimized PRM-OPDs exhibit an exceptional *EQE* of up to 2,484%, coupled with an impressively low *J_d_* of 6.63 × 10^−8^ A/cm^2^. Beyond mere photodetection, these devices uniquely enable direct visualization of light spot profiles and their dynamics in real time, establishing a novel dual-function modality for spot detection. Furthermore, we demonstrate the versatility of this concept by constructing a narrowband NIR PRM-OPD through the incorporation of an optical microcavity. This device achieves a spectral response from 700 to 850 nm, with a peak *EQE* of 499% at 780 nm and a notably low *J_d_* of 2 × 10^−8^ A cm^−2^ under a 20-V bias. This work opens a pathway for developing a new class of high-gain, low-dark-current, and multifunctional organic optoelectronic devices.

## Results and Discussion

**Fig. 1.**
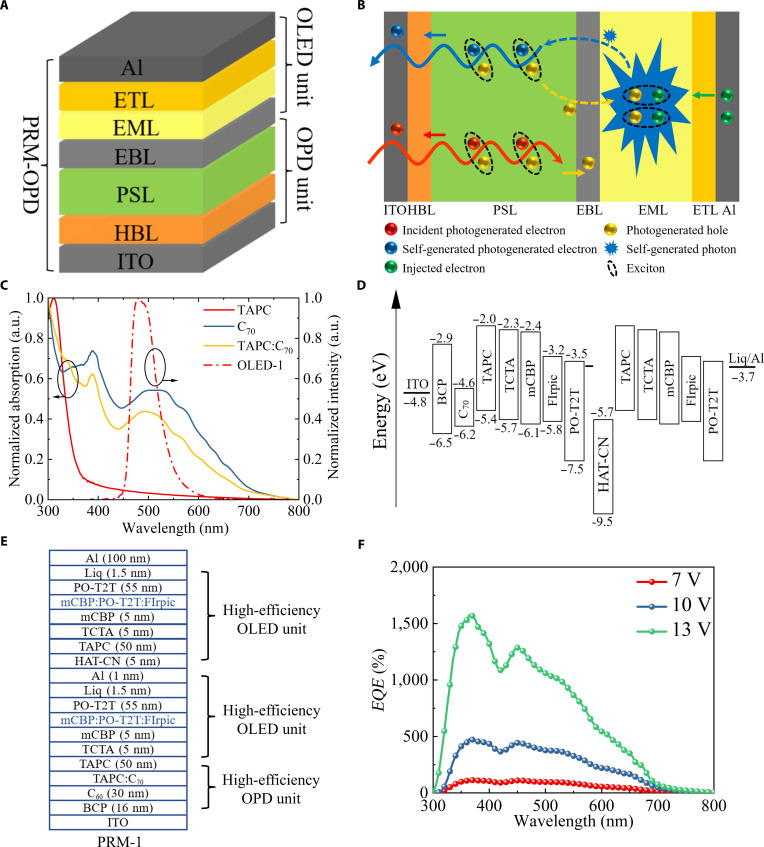
(A) Device structure of the photon-regeneration multiplication-type organic photodetector (PRM-OPD). (B) Working processes of the PRM-OPD under illumination and an applied bias. (C) Ultraviolet–visible (UV–Vis) absorption spectra of 1-bis[4-[*N*,*N*-bis(4-tolyl)amino]phenyl]-cyclohexane (TAPC), C_70_, and TAPC:C_70_ blend films and electroluminescence (EL) spectra of OLED-1. (D) Energy level diagram and (E) detailed device structure of the fabricated PRM-1. (F) external quantum efficiency (*EQE*) spectra of PRM-1 under different biases. ETL, electron transport layer; EML, emissive layer; EBL, electron blocking layer; PSL, photosensitive layer; HBL, hole blocking layer; ITO, indium tin oxide; OLED, organic light-emitting diode; OPD, organic photodetector; BCP, bathocuproine; TCTA, 4,4,4-tris(*N*-carbazolyl) triphenylamine; mCBP, 9,9′-biphenyl-3,3′-diylbis-9*H*-carbazole; FIrpic, iridium(III) bis[(4,6-di-fluorophenyl)-pyridinato-*N*,C2]; PO-T2T, ((1,3,5-triazine-2,4,6-triyl)tris(benzene-3,1-diyl))tris(diphenylphosphine oxide); HAT-CN, 1,4,5,8,9,11-hexaazatricarbon-hexacarbonitrile; Liq, lithium 8-hydroxyquinoline.

In this study, a PRM-OPD was formed by integrating an OLED unit with an OPD unit. The OLED unit was designed to supply additional photons. As depicted in Fig. 1A, the device structure consists of a hole blocking layer (HBL), a photosensitive layer (PSL), an electron blocking layer (EBL), an emissive layer (EML), and an electron transport layer (ETL) sandwiched between an indium tin oxide (ITO) anode and an aluminum (Al) cathode. The operational process in PRM-OPD under illumination and applied forward bias is illustrated in Fig. 1B. The PSL in the OPD unit absorbs incident photons to generate excitons, which then diffuse to the donor/acceptor interface, where they undergo separation into electrons and holes. The electrons are transported toward the anode, contributing to the photocurrent. The holes, however, are directed toward the OLED unit, where they recombine with electrons injected from the external circuit, leading to the generation of photons termed “self-generated photons”. Similar to incident photons, these self-generated photons can also be absorbed by the OPD unit, a process referred to as “feedback”, and converted into additional photocurrent, as well as new self-generated photons, through the aforementioned photoelectric conversion process. This phenomenon, which is termed as “photon regeneration”, allows for repeated photoelectric conversion under an applied bias, resulting in a significant gain in photocurrent. Notably, like most PM-OPDs, PRM-OPDs also involve external electron injection during the operational process. However, in PRM-OPDs, these injected electrons contribute to the gain by generating self-generated photons. To understand this process from the perspective of the circuit, we constructed an equivalent circuit model (Fig. S1, Supplementary Materials), in which the PRM-OPD can be represented as a photodetector connected in series with the OLED. This model intuitively shows that only when a closed circuit is formed under illumination, and the OPD drives the OLED to emit light, can the self-generated photon-based cyclic gain process be initiated. The externally injected electrons are a necessary condition for maintaining the integrity of the circuit and the photon-regeneration cycle, but they themselves do not directly provide gain. In the absence of illumination, the electrons injected by the external circuit are hindered at the interface between the EBL and the EML within the OLED unit, while the holes are obstructed at the interface between the electrode and the HBL within the OPD unit. Consequently, the dark current is effectively suppressed. Obviously, the PRM-OPD uses external electrical energy to drive OLED emission, thereby establishing and maintaining a “photon–electron–photon” positive feedback loop. This loop enables a single incident photon to be converted into multiple collected photogenerated electrons, thereby achieving photoelectric current gain. From an energy perspective, all of the additional energy required for the gain undoubtedly comes from the external power source. The entire system macroscopically follows the principle of energy conservation between input and output.

To fabricate highly efficient PRM-OPDs, a well-established PSL composed of the classical 1-bis[4-[*N*,*N*-bis(4-tolyl)amino]phenyl]-cyclohexane (TAPC):fullerene C_70_ bulk heterojunction for the OPD unit was utilized [38]. The absorption spectra depicted in Fig. 1C clearly show that the TAPC:C_70_ blend film exhibits prominent absorbance within the ultraviolet–visible region. In this configuration, fullerene C_70_ and bathocuproine (BCP) are utilized as the HBL, and TAPC plays the dual roles of EBL and effective connection of the OPD and OLED units. To achieve efficient photocurrent gain through improving the utilization of self-generated photons, 2 key factors are essential. Firstly, the electroluminescence (EL) spectra of the OLED unit and the absorption spectra of the OPD unit should overlap as much as possible. This will maximize the probability of the photons emitted by the OLED being absorbed by the OPD to generate a photocurrent. Secondly, the OLED unit needs to exhibit high electron–photon conversion efficiency, ensuring that a large number of electrons are effectively converted into photons for subsequent recycling and utilization in the OPD unit. Therefore, a blue phosphorescent OLED unit was adopted, where the EML was fabricated by blending the electron donor material 9,9′-biphenyl-3,3′-diylbis-9*H*-carbazole (mCBP) with the electron acceptor material ((1,3,5-triazine-2,4,6-triyl)tris(benzene-3,1-diyl))tris(diphenylphosphine oxide) (PO-T2T) to form an exciplex as the host, while the blue phosphorescent material iridium(III) bis[(4,6-di-fluorophenyl)-pyridinato-*N*,C2] (FIrpic) was employed as the dopant. Additionally, PO-T2T was utilized as the ETL, and 4,4,4-tris(*N*-carbazolyl) triphenylamine (TCTA) and mCBP were employed as EBLs to connect the TAPC layer of OPD units. By gradually decreasing the energy levels of the highest occupied molecular orbitals (HOMOs) of TAPC, TCTA, and mCBP, the energy losses are minimized during hole transport [39]. Furthermore, to enhance the electron-to-photon conversion efficiency, a 1,4,5,8,9,11-hexaazatricarbon-hexacarbonitrile (HAT-CN)/TAPC planar heterojunction was used to connect 2 OLED units, achieving a tandem OLED (T-OLED) configuration. Here, we prepared T-OLED (designated as OLED-1), with the device structure shown in Fig. S2 (Supplementary Materials). The chemical structures of the materials used are shown in Fig. S3 (Supplementary Materials). The EL spectrum of OLED-1 covered a wavelength range from 450 to 550 nm (Fig. 1C), which aligns well with the absorption spectrum of the OPD unit. The energy levels and detailed device structure of PRM-OPD-1 (referred to as PRM-1) prepared by integrating the OPD unit and the OLED unit are shown in Fig. 1D and E, respectively. A more detailed description of the flow of electrons and holes, as well as the generation of self-generated photons in the dark and under illumination based on the device energy band, is provided in Fig. S4 (Supplementary Materials). As shown in Fig. 1F, the peak *EQE* value of PRM-1 is as high as 1,578% under a 13-V bias, confirming that this type of structure can achieve the gain effect.

**Fig. 2.**
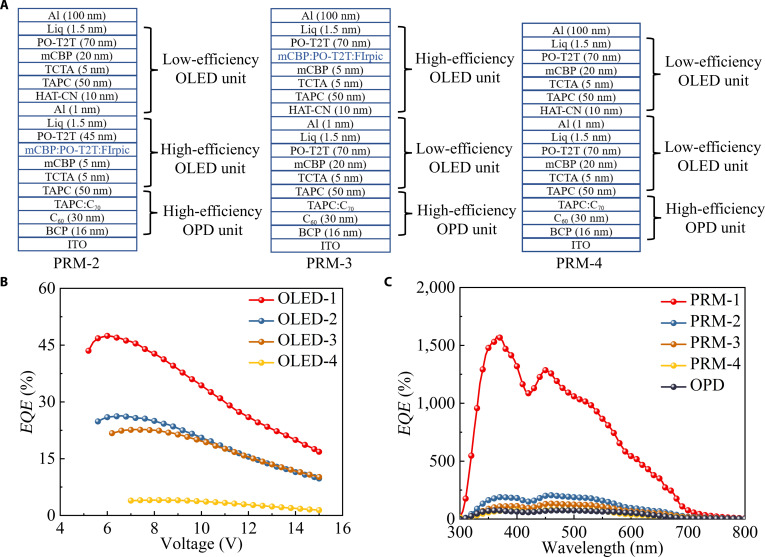
(A) Detailed structures of PRM-2, PRM-3, and PRM-4. (B) *EQE*–voltage characteristics of OLED-1, OLED-2, OLED-3, and OLED-4. (C) *EQE* spectra of PRM-1, PRM-2, PRM-3, and PRM-4 under a 13-V bias and OPD-1 under an 8-V bias.

To investigate the influence of OLED unit efficiency on the performance of the PRM-OPD, we prepared PRM-OPD devices (named PRM-2, PRM-3, and PRM-4) by integrating additional T-OLED units on the basis of different light-emitting layer structures and positions and simultaneously prepared the reference device OPD-1. The detailed structures of the PRM-2, PRM-3, and PRM-4 devices are provided in Fig. 2A. The structure of the conventional reference device OPD-1 is detailed in Fig. S5 (Supplementary Materials). Corresponding T-OLEDs, denoted as OLED-2, OLED-3, and OLED-4, were also fabricated to provide a visual representation, as shown in Fig. S6 (Supplementary Materials). The EL spectra of OLED-2, OLED-3, and OLED-4 cover a wavelength range from 450 to 550 nm (Fig. S7, Supplementary Materials), which is almost identical to that of OLED-1. Thus, the influence caused by different spectra can be eliminated. From Fig. 2B, the maximum *EQE*s of OLED-1, OLED-2, OLED-3, and OLED-4 are 47.5%, 26.3%, 22.7%, and 4.1%, respectively. Under a 13-V bias, the peak *EQE*s of PRM-2, PRM-3, and PRM-4 reach 202%, 132%, and 80%, respectively, which are all higher than the 78% of OPD-1 but still observably lower than the corresponding value of PRM-1. More importantly, the *EQE* of the PRM-OPD shows a significant growth trend with the improvement of OLED unit efficiency, demonstrating a clear positive dependency. Moreover, as shown in Fig. S8 (Supplementary Materials), the *J_d_* values of all PRM-OPD devices are stable at a level of 10^−8^ A/cm^2^, which is much lower than that of OPD-1.

**Fig. 3.**
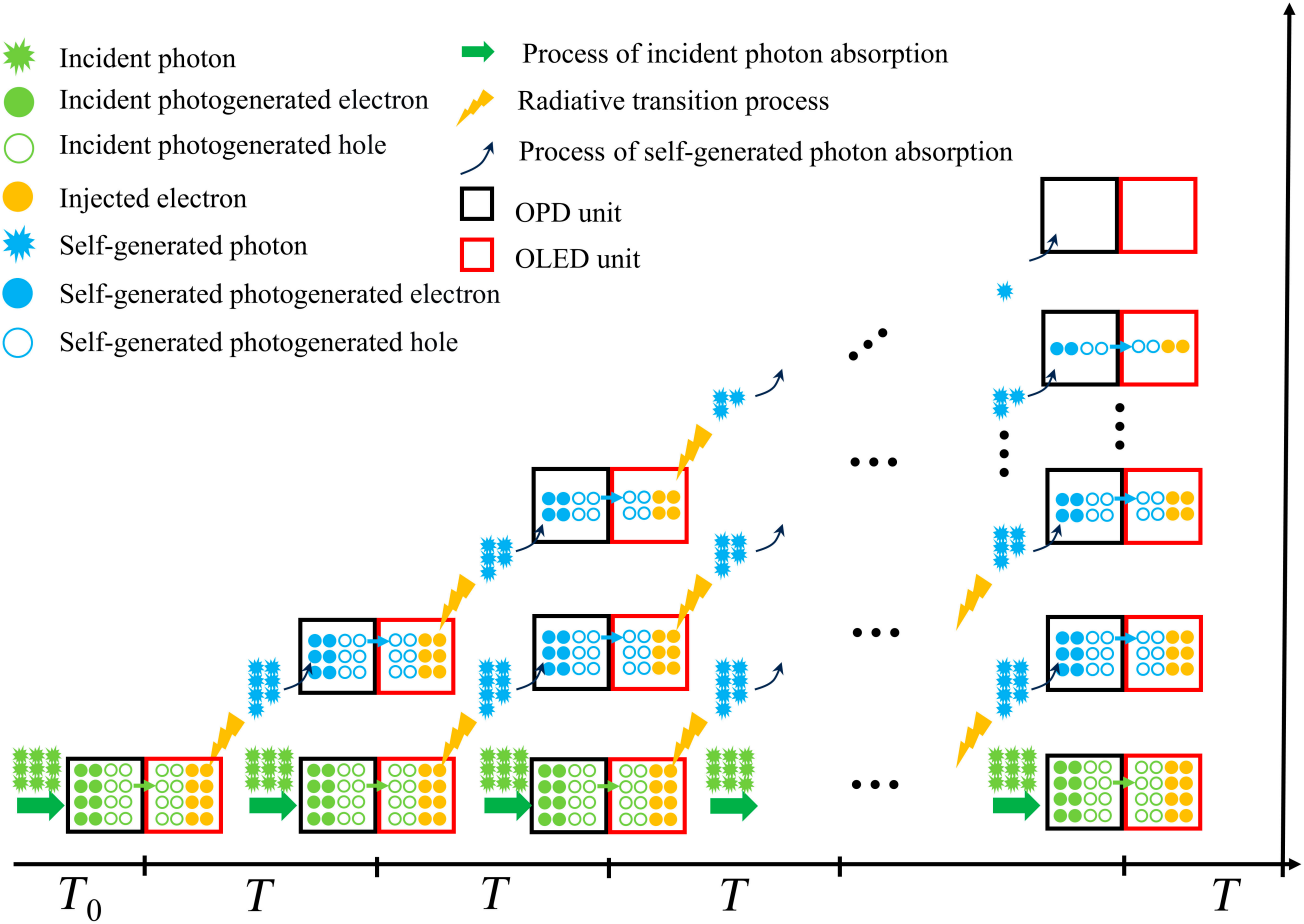
Simple example diagram of the working process in the PRM-OPD under constant illumination and an applied bias.

To elucidate their difference in device performance, the situation from the onset of light illumination to a stable photocurrent response is considered. In the initial phase after illumination (*T*_0_), the photocurrent is generated solely by the absorption in the OPD unit due to the lack of feedback, similar to the response of a non-gain OPD. Under an applied external electric field, the photogenerated electrons in the OPD unit are collected by the anode, while the photogenerated holes are injected into the EML of the OLED unit, leading to the emission of the OLED unit. A portion of these generated photons from the OLED unit are then absorbed by the OPD unit to again produce a photocurrent in the OPD unit. This is a cyclic process. Over time, photons will be continuously generated and then absorbed, leading to the recycling reuse of photons. Generally speaking, the response time of an OPD unit is shorter than the EL decay time of a phosphorescent OLED unit, indicating that the duration of one cycle iteration primarily depends on the emission time of the OLED unit. Here, we define the interval period between 2 emissions as *T* and discuss the magnitude of the photocurrent when the device reaches a steady-state equilibrium. Figure 3 presents a streamlined schematic diagram of the iterative process in the PRM-OPD. Here, the horizontal axis represents the time required for different numbers of feedback iterations, with intervals of one period *T*, while the vertical axis represents the photocurrent formed by different numbers of feedback iterations. The photocurrent at the bottom of the vertical axis originates from the continuous irradiation of external light. Typically, the feedback process involves photon losses, and the photocurrent gradually decreases after multiple iterations. However, the photocurrent at any given moment is the cumulative result of continuous incident light in different feedback iterations. Based on previous calculation, the photocurrent can be approximated as the sum of a geometric series (supporting information in a previously published article) [35]. Since the common ratio is generally less than 1, the sum of the geometric series converges. Therefore, under illumination, the device will form a stable photocurrent, allowing for the calculation of device *EQE*. In order to facilitate the analysis of the gain in the photon-regeneration device, the formula to calculate the *EQE* can be written as follows:

$$\mathrm{EQE}=\frac{\eta_{A}\eta_{\mathrm{PSL}}}{1-{\alpha \eta }_{\mathrm{PSL}}\eta_{\mathrm{EML}}}$$ (1)

where *η*_A_ represents the light absorption efficiency in the PSL. *η*_PSL_ corresponds to the efficiency of converting light into current within the PSL. Their product, *η*_A_*η*_PSL_, represents the *EQE* contributed by the OPD unit in absorbing incident light in *T*_0_. *η*_EML_ denotes the efficiency of converting a carrier into a photon in the OLED unit. *α* is defined as the proportion of self-generated photons absorbed by the PSL. Considering that the device contains 2 OLED units, *α*_1_ and *α*_2_ are introduced to represent the self-generated photon absorption ratios in the PSL originating from EML1 and EML2 in T-OLED units, respectively. Here, EML1 is the EML closer to the OPD unit, while EML2 is the one farther from the OPD unit. Therefore, the *EQE* of PRM-OPDs containing T-OLED units can be expressed by Eq. (2) [35]:

$$\mathrm{EQE}=\frac{\eta_{A}\eta_{\mathrm{PSL}}}{1-\left(\alpha_{1}\eta_{\mathrm{EML}1}+\alpha_{2}\eta_{\mathrm{EML}2}\right)\eta_{\mathrm{PSL}}}$$ (2)

All PRM-OPDs have the same OPD unit and similar EL spectra, so their difference mainly lies in the efficiency of carrier conversion into photons in the OLED unit. In addition, compared with a PRM-OPD with a TAPC:C_60_ blend film as the PSL, the *EQE* of PRM-1 is much larger due to the higher efficiency of photon conversion into photocurrent in the OPD unit (Fig. S9 in the Supplementary Materials).

**Fig. 4.**
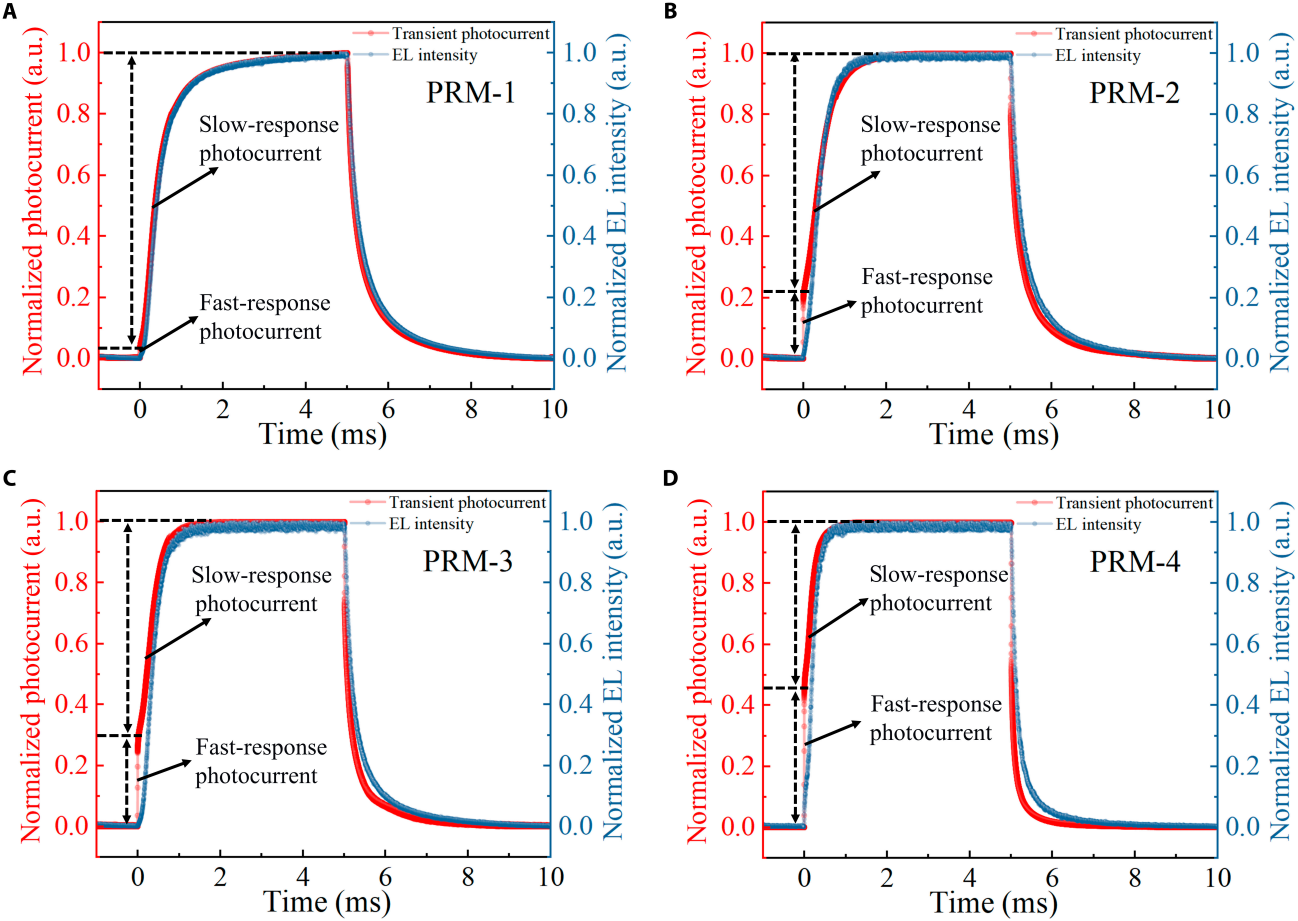
Transient photocurrent response curves (*J*–*t*) and EL curves of (A) PRM-1, (B) PRM-2, (C) PRM-3, and (D) PRM-4 under an on state at a pulse frequency of 100 Hz and a 13-V bias measured by LED illumination with a peak of 520 nm.

Based on the above concept, we can observe the light emission of the device during its operation. Experimentally, during the transient photoresponse testing of the device, we simultaneously used a photomultiplier tube to observe the changes in device intrinsic light emission. As illustrated in Fig. 4, the growth trajectory of photocurrent can be distinctly divided into 2 phases, which align well with the *T*_0_ and *T* stages outlined in Fig. 3. As expected, when light instantly illuminates the device, the photocurrent exhibits a rapid response, similar to that of non-gain devices. Conversely, during the *T* phase, the light emitted from the device itself, detected by a photomultiplier tube, gradually intensifies and stabilizes, consistent with the photocurrent enhancement. Additionally, the ratio between the fast-response photocurrent and the slow-response photocurrent is also consistent with the ratio of the *EQE*s of the non-gain device and gain device.

**Fig. 5.**
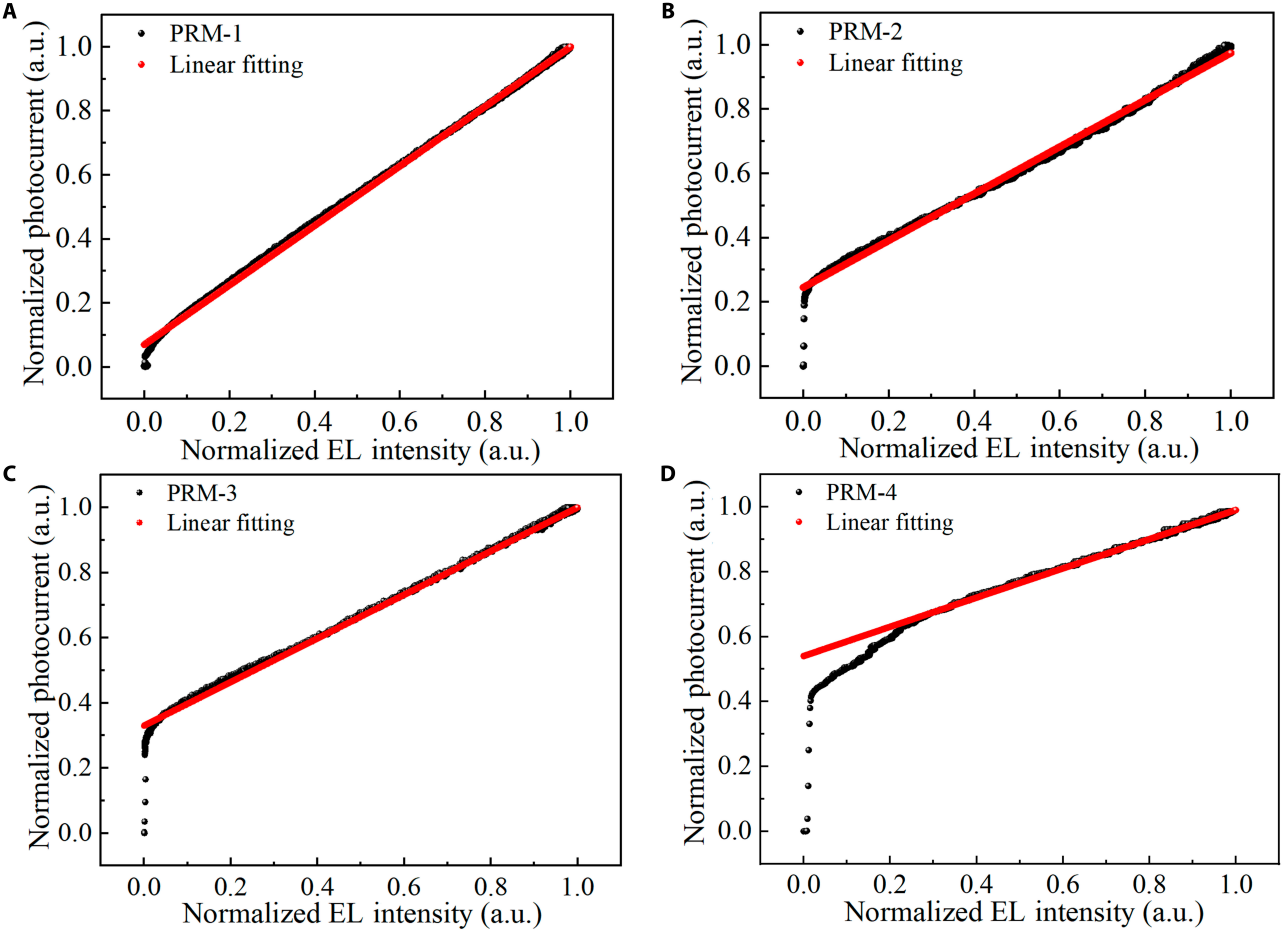
Characteristics and linear fitting curves of EL intensity–photocurrent for (A) PRM-1, (B) PRM-2, (C) PRM-3, and (D) PRM-4 in the ascending phase.

Specifically, when light starts to illuminate but before forming optical feedback and the EL intensity of the 4 devices is zero, the rapid rise in photocurrent further confirms the anticipated operational process during the *T*_0_ stage, which is a short period of time close to the zero point. As the feedback process initiates and the EL intensity increases, the photocurrent also starts to increase. More importantly, as illustrated in Fig. 5 and Fig. S10 (Supplementary Materials), based on the mutual conversion relationship between photocurrent and self-generated photons, a linear fitting is performed on the second half of the rising and falling stages in the transient photocurrent response and EL decay curves, confirming the existence of the feedback process. This consistency, as demonstrated by the above transient response experiments, validates the rationality of our proposed working mechanism. Evidently, the proportions of photocurrent generated by these devices in *T*_0_ are approximately 4%, 22%, 30%, and 45%, respectively. Based on these ratios, it is calculated that the *EQE* values directly generated by the PSL absorbing incident photons are 41%, 39%, 37%, and 34%, respectively. Under the same bias voltage of 13 V, the voltage division across the OPD unit varies due to the distinct turn-on voltages of OLED units (5.2, 5.8, 6.2, and 7 V, respectively), thereby resulting in different *EQE* values directly generated by the PSL. Further, as shown in Fig. S11 (Supplementary Materials), the *EQE* of OPD-1 increases with the increase in applied voltage and eventually reaches saturation under a high bias. At this saturation point, the conversion efficiency *η*_PSL_ approaches unity (*η*_PSL_ ≈ 1). Concurrently, based on established literature reports ^[^^40^^]^, the emission layer efficiency (*η*_EML_) of the implemented light-emitting unit is also approximated as 1. Under these conditions, we derive the following quantitative relationships: *α*_1_ ≈ 0.57 and *α*_2_ ≈ 0.38, as detailed in Note S1. In addition, the transient photocurrents of these devices can be exponentially fitted (Fig. S12, Supplementary Materials), which is almost the same pattern as Eq. (2), proving the accuracy of Eq. (2). Based on the fitting equation, the period *T* values of these devices are calculated to be 21, 89, 93, and 103 μs, respectively (Note S2). Their differences are attributed to the rise times of the OLED (Fig. S13, Supplementary Materials), that is, the times for the photocurrent to increase from 10% to 90% of its steady-state value, which are 48, 115, 118, and 180 μs, respectively. Obviously, the time required for these devices to reach their maximum is determined by the photoelectric conversion efficiency of the OLED unit and the time of rise.

**Fig. 6.**
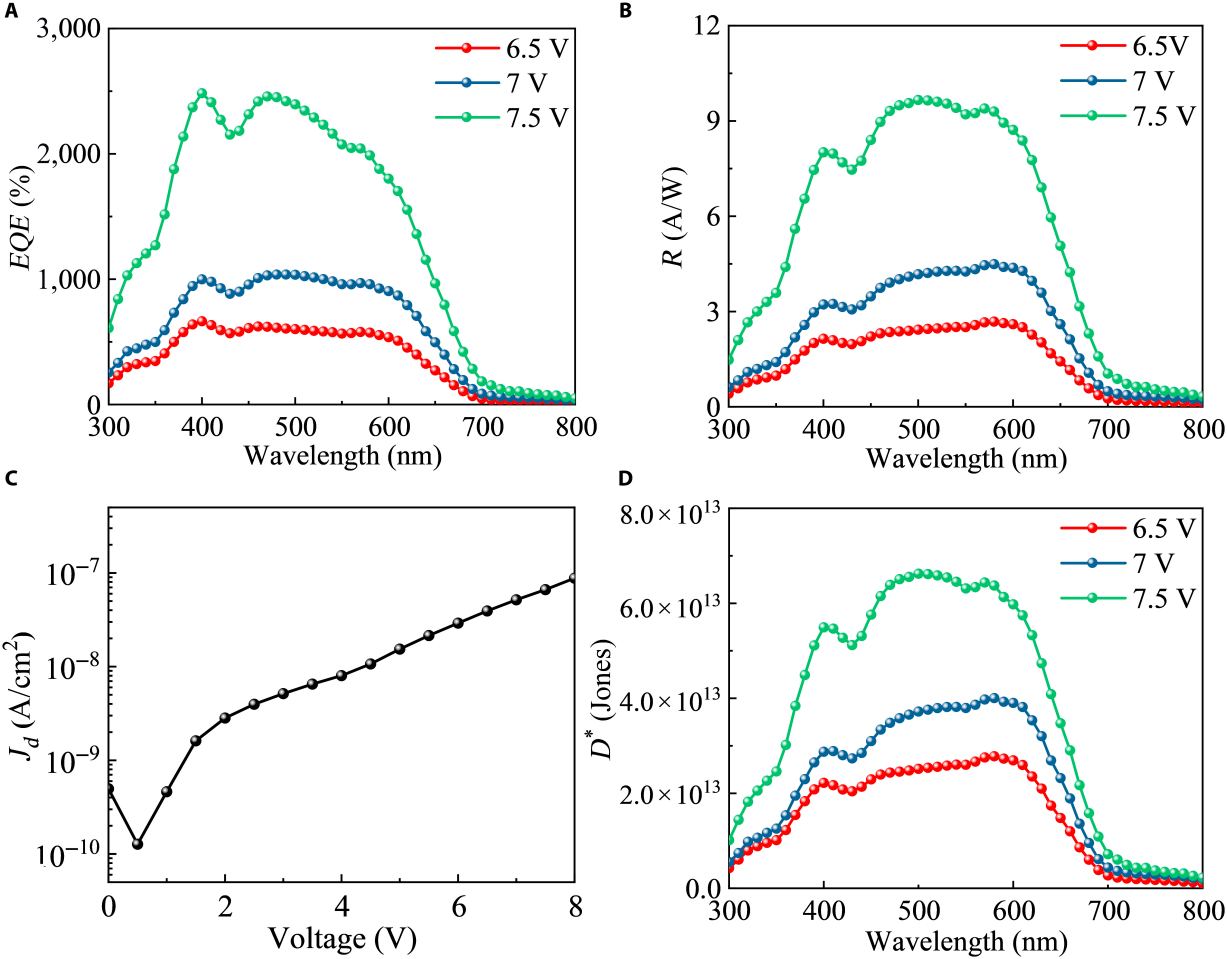
(A) *EQE*, (B) *R*, (C) dark current density–voltage (*J_d_*–*V*), and (D) D* characteristics of PRM-5 under different bias voltages.

By reducing the thickness of the hole transport layer to close the emission layer in the OLED unit to the PSL in the OPD unit, it is expected to further enhance *EQE* by enhancing the absorption rate *α* of the active layer for self-generated photons. The thickness of the ETL is also adjusted to balance carrier transport within the OLED unit, thus improving *η*_EML_. Furthermore, the concentration of TAPC in the PSL is reduced from 35% to 15%, a modification that effectively boosts the exciton dissociation efficiency, thus increasing *η*_PSL_ [39]. To further suppress the dark current, radio-frequency magnetron sputtering technology is employed to deposit zinc oxide (ZnO) with a lower HOMO level, replacing BCP as the hole barrier layer (the energy level diagram is shown in Fig. S14, Supplementary Materials). The refined device structure (named PRM-5) is illustrated in Fig. S15 (Supplementary Materials). As demonstrated in Fig. 6A, PRM-5 achieves a notably higher *EQE* of 2,484% at a reduced bias voltage of 7.5 V, which is higher than that of PRM-1.

The responsivity (*R*) characteristics are calculated according to Eq. (3):

$$R=\frac{\mathrm{EQE}\times \mathrm{hv}}{q}$$ (3)

where *hv* is the energy of the incident photon in electron-volts and *q* is the electron charge. As shown in Fig. 6B, the maximum *R* of PRM-5 reaches 9.65 A/W at 500 nm under a 7.5-V bias. The current density (*J_d_*)–voltage (*V*) curve of PRM-5 is shown in Fig. 6C; the dark current density is 6.64 × 10^−8^ A/cm^2^ under a 7.5-V bias. The specific detectivity (D*), which indicates the ability to detect weak incident optical power, is also an important parameter of OPDs. On the assumption that the total noise current is dominated by the shot noise component in dark current, D* can be calculated by Eq. (4). As illustrated in Fig. 6D, D* reaches a maximum value of 6.62 × 10^13^ Jones at 500 nm under a 7.5-V bias.

$$D^{*}=\frac{R}{\sqrt{2{\mathrm{qJ}}_{d}}}$$ (4)

In order to obtain the actual noise value of the device, we measured the noise current (*i_n_*) using a low-noise current amplifier (DLPCA-200, Femto) and a digital oscilloscope (Keysight, DSOS404A), and the results are shown in Fig. S16 (Supplementary Materials). Therefore, the actual D* can be calculated according to the following formula:

$$D^{*}=\frac{\sqrt{A}}{\mathrm{NEP}}=\frac{R\sqrt{\mathrm{AB}}}{i_{n}}\;\left(\mathrm{cm}\;{\mathrm{Hz}}^{1/2}\;W^{-1}\right)$$ (5)

where *A* is the active area of 0.16 cm^2^, *B* is the electrical bandwidth, and *i_n_* is the total noise current. The *i_n_* value is about 1.43 × 10^−13^ A Hz^−1/2^ at a modulation frequency of 20 Hz under a 7.5-V bias, and the corresponding D^*^ value is 2.69 × 10^13^ Jones under 500-nm light illumination.

We conducted tests to evaluate the transient response speed of PRM-5 under different biases. The rise time, denoted as *τ*_r_, is defined as the period during which the photocurrent increases from 10% to 90%. Conversely, the fall time, denoted as *τ*_f_, signifies the duration over which the photocurrent decreases from 90% to 10%. Figure S17 (Supplementary Materials) presents the normalized transient response of PRM-5 under different biases. Obviously, *τ*_r_ and *τ*_f_ increase with the increase in bias voltage. The *τ*_r_ and *τ*_f_ of PRM-5 are 19.5 and 15.7 ms, respectively.

It is important for photodetectors to exhibit constant responsivity from weak to strong light for practical applications. As depicted in Fig. S18 (Supplementary Materials), we thus characterized the photocurrent of PRM-5 under an applied voltage of 7.5 V and incident white light intensities from 10^−9^ to 10^−5^ W cm^−2^. The photocurrent increases with the light intensity across about 4 orders of magnitude.

**Fig. 7.**
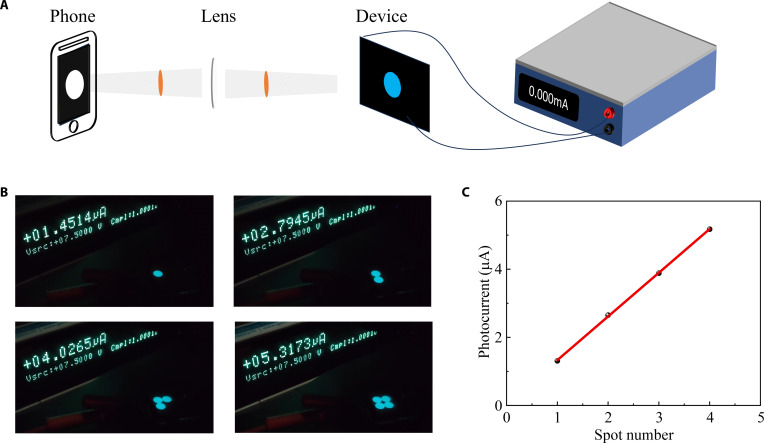
(A) Schematic diagram of the PRM-OPD detection light source with an area of 280 mm^2^. (B) Example diagram of the device surface with different numbers of circular spots under radiation. (C) Characteristic curve of photocurrent–spot number.

To demonstrate the dual-functional working state of the designed devices, we further fabricated a PRM-OPD with an area of 280 mm^2^ (Fig. S19 in the Supplementary Materials) and used it to detect the size and shape of the faint light signals. As illustrated in Fig. 7A, the light spot emitted from a mobile phone is projected onto the PRM-OPD through a lens, and the photocurrent can be measured using a 2400 sourcemeter. As shown in Fig. 7B, the different numbers of circular light spots can be directly observed on the PRM-OPD through projection, which reveals both the quantity and shape of the spots. As depicted in Fig. 7C, the photocurrent generated by the PRM-OPD is proportional to the number of light spots. The real-time display video of the light spots can be viewed in Movie S1 in the Supplementary Materials.

**Fig. 8.**
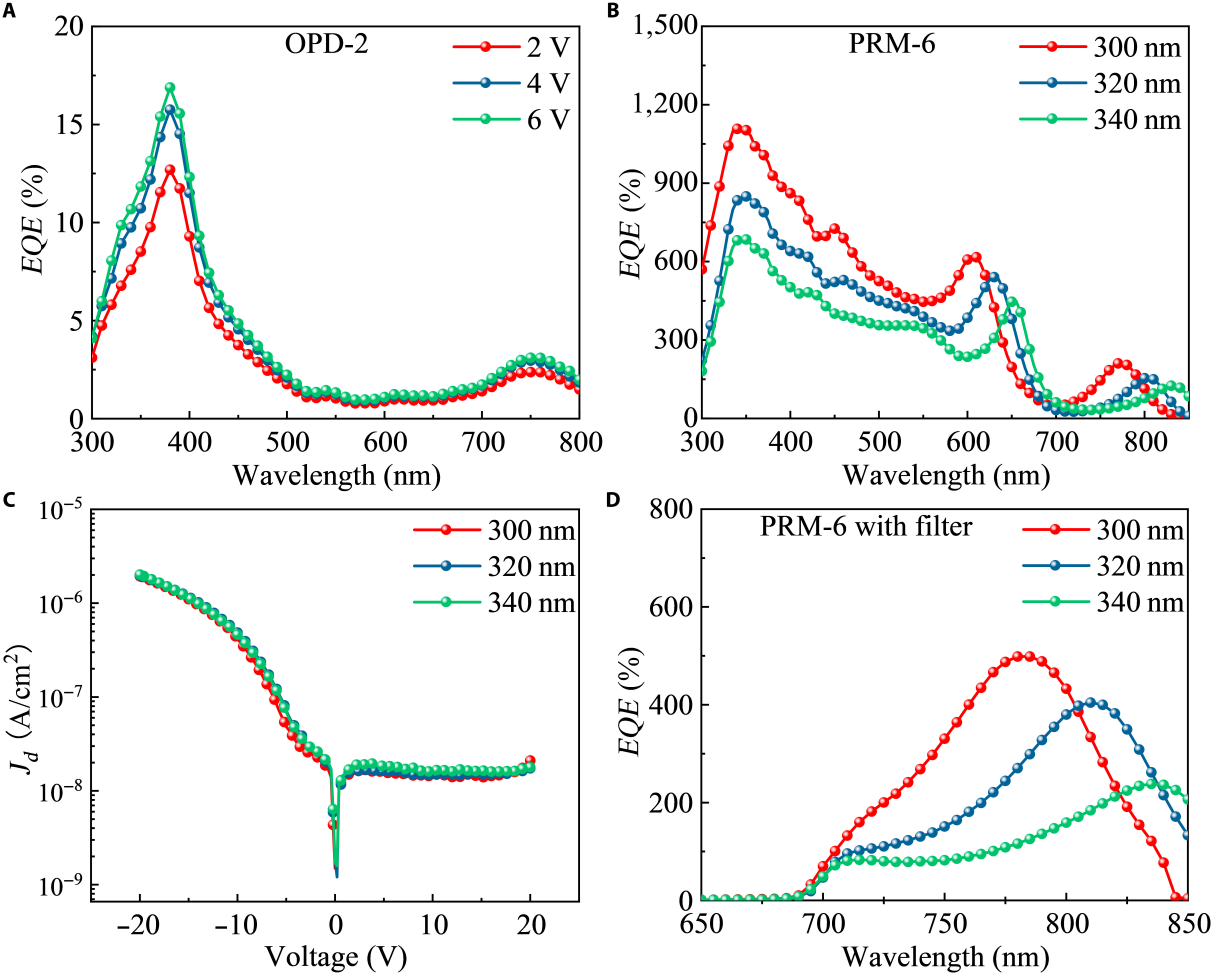
(A) *EQE* spectra of OPD-2 under different biases. (B) *EQE* spectra and (C) dark current density–voltage curves of PRM-6 for different thicknesses of PSL from 300 to 340 nm. (D) *EQE* spectra of PRM-6 with filter for different thicknesses of PSL from 300 to 340 nm.

Extending the response spectrum of the OPD to the NIR region is also necessary, generally including the use of narrow-bandgap materials as the optical active layer and the enhancement of the charge-transfer (CT) state response by the microcavity effect [41]. Due to the limitation of organic small-molecule materials available that exhibit absorption in the NIR range, we integrated partially transmissive and fully reflective Al mirror electrodes together to establish an optical resonant microcavity and fabricated PRM-OPD-6 (referred to as PRM-6). As a contrast, a reference OPD (referred to as OPD-2) was prepared. The detailed device structures are depicted in Fig. S20 (Supplementary Materials). This microcavity enhances the absorption of CT excitons between TAPC and C_70_. As depicted in Fig. 8A, OPD-2 exhibits only a weak response in the range of 700 to 800 nm with a maximum *EQE* of 3%. However, as shown in Fig. 8B, the spectral response of PRM-6 undergoes a redshift to 850 nm as the PSL thickness increases from 300 to 340 nm, and the device with a PSL thickness of 300 nm exhibits superior performance under a bias voltage of 23 V. Notably, it achieves a maximum *EQE* of 220% in the NIR region, a value that is 73 times greater than that of OPD-2. Additionally, as shown in Fig. 8C, the dark current of PRM-6 remains below 2 × 10^−8^ A/cm^2^ at a 20-V bias. Furthermore, in order to detect only the NIR wavelength and utilize the emitting light of the integrated OLED unit, a fixed filter at the bottom end of PRM-6 was incorporated, thus increasing the absorption of the OPD unit for self-generated photons. As depicted in Fig. 8D, the device demonstrates a narrowband response in the NIR region, with a full width at half maximum of approximately 75 nm. Remarkably, a maximum *EQE* of 499% is achieved in the NIR region, outperforming OPD-2 by a factor of 166 times, demonstrating the universality of the designed device structure.

## Conclusion

Based on the principle of photon regeneration, we designed and fabricated a novel dual-function PM-OPD. By integrating OPDs with different OLED units, we fabricated a series of PRM-OPDs to verify the feasibility of the working principle. This gain mechanism was further confirmed through the transient photocurrent response and luminescence of the devices. By combining the quantitative analysis results from these transient measurements, we optimized the device performance, which exhibits a high *EQE* of 2,484% and an extremely low dark current density of 6.63 × 10^−8^ A cm^−2^ at a bias of 7.5 V. The rise and fall times of the device are 19.5 and 15.7 ms, respectively. Currently, the speed is primarily limited by the intrinsic phosphorescent emission lifetime, which may be overcome by an efficient hybrid system of fluorescence and phosphorescence or a fluorescence-sensitized phosphorescence system with a short emissive lifetime. Moreover, it can be seen that the devices can simultaneously achieve visualization of spot morphology and detection of light intensity, demonstrating a dual-function working mode. As a versatile gain design structure, the aforementioned gain structure can also be used to address the issue of a low *EQE* in conventional microcavity-enhanced CT state exciton absorption devices, which is caused by low light absorption efficiency. Compared to conventional microcavity devices, which typically exhibit an *EQE* of around 3% at 780 nm, the optimized photon-regeneration gain device demonstrates a remarkably high *EQE* of 499% and, more impressively, also exhibits an extremely low dark current density, providing a new idea for the development of high-performance PM-OPDs.

## Materials and Methods

### Materials and devices

Fullerene-C60 (C_60_), fullerene-C70 (C_70_) and BCP were purchased from Sigma-Aldrich, and TAPC, TCTA, FIrpic, mCBP, PO-T2T, HAT-CN, and lithium 8-hydroxyquinoline (Liq) were purchased from Jilin OLED; all materials were used without any further purification. PRM-OPDs were prepared by depositing 140-nm ITO on the glass surface as the substrate. Microcavity devices were fabricated by directly depositing materials on glass substrates. All substrates were treated by O_2_-plasma treatment for 4 min to further clean the surface.

The subsequent layers were composed of organic materials, and metals were deposited via thermal evaporation under controlled high-vacuum conditions with a base pressure of ≈10^−5^ Pa. The organic materials (BCP, TAPC, TCTA, mCBP, PO-T2T, HAT-CN, and Liq) were evaporated at rates of 1.5 Å/s, C_60_ and C_70_ were deposited at 3.5 Å/s, and the metal electrodes were evaporated at 3 Å/s. For the active layer, after setting the evaporation rate of C_70_, the rate of TAPC was adjusted according to the donor–acceptor ratio. Similarly, in the emitting layer, following the determination of the evaporation rates for mCBP and PO-T2T, the evaporation rate of FIrpic was controlled based on the host–guest ratio. Evaporation rates, layer thicknesses, and mixing ratios were controlled in situ via quartz crystal microbalances, and the thickness was calibrated by using a Dektak 6M profiler (Veeco). The geometrical intersection of the top Al or ITO electrode and the bottom Al electrode defines the photoactive area of 20 mm^2^, and the metal electrodes were deposited through a shadow mask. All measurements were carried out under packaging conditions.

### Characterization

A setup by 7-Star Optical Instruments Co., Ltd, Beijing, was used for *EQE* spectral measurements. The incident light from a halogen lamp (250 W) passing through a monochromator was chopped at 35 Hz and focused on the active area of the devices. The photocurrent signal was first amplified using a low-noise current amplifier (DLPCA-200, Femto) and then detected by a lock-in amplifier (SR830, Stanford Research Systems). The reverse biases on the devices were obtained using a Keithley 236 Source-Measure Unit. A crystalline silicon photodiode (S1337-1010BQ, Hamamatsu) calibrated by the National Institute of Metrology of China was used as a reference before each measurement. For dark current measurement, the current–voltage characteristics were recorded using a Keithley 2636B source measurement unit. The photodetectors were kept in a dark box during the measurements. The absorption spectra were recorded by using a Shimadzu UV-3600 spectrophotometer.

## Data Availability

The data that support the findings of this study are available from the corresponding authors upon reasonable request.

## Supplementary Materials

Supplementary 1 Notes S1 and S2 Figs. S1 to S19 Table S1 Movie S1
