# Supplementary material for: A Novel Dual-Functional Photomultiplication-Type Organic Photodetector with Photon-Regeneration Gain by Integrated Organic Light-Emitting Diodes
Source: Research (Wash D C). 2026 Jan 26;9:1094. doi: 10.34133/research.1094 (PMC12833821; doi:10.34133/research.1094)
Supplement: Supplementary 1 — Notes S1 and S2 Figs. S1 to S19 Table S1 Movie S1 [file research.1094.f1.zip › Supporting Information(Clean Version with Author Info).docx]

Supplementary Materials for

**A novel dual-functional photomultiplication-type organic photodetectors with photon-regeneration gain by integrated organic light-emitting diodes**

Ji Li, Liqing Yang, Guo He, Jinghao Fu, Rentao Dong, Dechao Guo, Dezhi Yang*, and Dongge Ma*

Institute of Polymer Optoelectronic Materials and Devices, Guangdong Basic Research Center of Excellence for Energy & Information Polymer Materials, Guangdong-Hong Kong-Macao Joint Laboratory of Optoelectronic and Magnetic Functional Materials, State Key Laboratory of Luminescent Materials and Devices, South China University of Technology, Guangzhou 510640, China

E-mail: [msdzyang@scut.edu.cn](mailto:msdzyang@scut.edu.cn), and [msdgma@scut.edu.cn](mailto:msdgma@scut.edu.cn)

**This PDF file includes:**

Table S1. Key Parameters of Representative Broadband PM-OPDs over the past five years

Figure S1. Equivalent circuit diagram of PRM-OPDs.

Figure S2. The detailed structures of OLED-1.

Figure S3. The chemical structure of the materials used.

Figure S4. The working mechanism diagrams of the device a) under illumination and b) in the dark state, constructed based on the energy levels of the device.

Figure S5. The detailed device structures of OPD-1.

Figure S6. The detailed structures of OLED-2,3,4.

Figure S7. The EL spectra of OLED-1, 2,3,4.

Figure S8. The dark current density-voltage curves of PRM-1, 2, 3, 4 and OPD-1.

Figure S9. a) The detailed structures of OPD and PRM with TAPC:C_60_ blend films as the PSL. b) UV–vis absorption spectra of TAPC:C_60_ and TAPC:C_70_ blend films. *EQE* spectra of c) OPD and d) PRM with TAPC:C_60_ and TAPC:C_70_ blend films as the PSL, respectively.

Figure S10. Characteristics and liner fitting curves of EL intensity-photocurrent for a) PRM-1, b) PRM-2, c) PRM-3, and d) PRM-4 in the descending phase, respectively.

Figure S11. The characteristics of *EQE –* *V* of OPD-1 under different wavelengths of light irradiation.

Figure S12. The transient photocurrent characteristics and exponential fitting curves of a) PRM-1, b) PRM-2, c) PRM-3, and d) PRM-4 in the ascending phase, respectively.

Figure S13. The transient photocurrent characteristics and exponential fitting curves of a) OLED-1, b) OLED-2, c) OLED-3, and d) OLED-4 in the ascending phase, respectively.

Figure S14. The energy level diagrams of BCP and ZnO.

Figure S15. The detailed structure of PRM-5 and OPD-2 integrated device.

Figure S16. Measured noise spectra of the PRM-5 under 7.5V bias. The dashed line is the shot noise calculated from dark current.

Figure S17. The transient photocurrent responses (*J–t* curves) of PRM-5 at a pulse frequency of 100 Hz with a device area of 0.2 cm^2^ measured by LED illumination with a peak of 520 nm under a) 6.5 V, b) 7 V and c) 7.5 V bias, respectively.

Figure S18. Incident light intensity-photocurrent density of PRM-5 under 7.5 V.

Figure S19. The schematic diagrams of large area PRM-OPD operation under dark state (left) and fluorescent illumination (right).

Figure S20. The detailed structures of PRM-6 and OPD-2.

Supplementary Note 1: $\begin{matrix} \alpha_{1}\approx0.57 \\ \alpha_{2}\approx0.38 \end{matrix}$

Supplementary Note 2: $T=\frac{\ln0.96}{-1.95}\approx21\mu s$

Table S1. Key Parameters of Representative Broadband PM-OPDs over the past five years

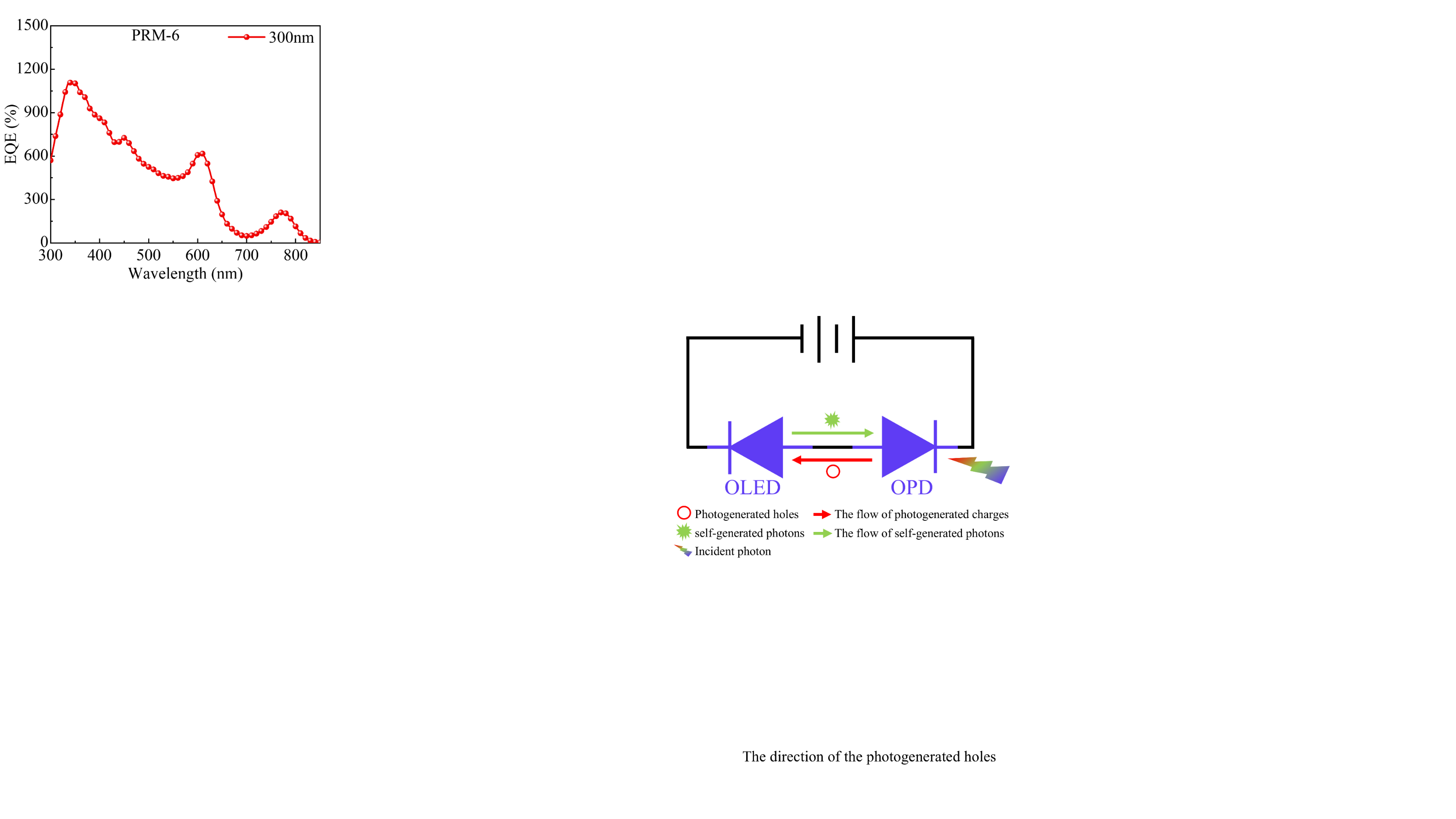


Figure S1. Equivalent circuit diagram of PRM-OPDs.


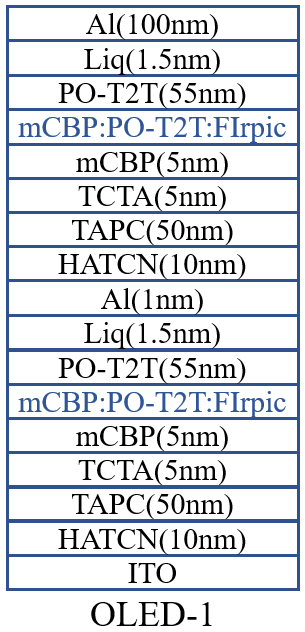


Figure S2. The detailed device structures of OLED-1.


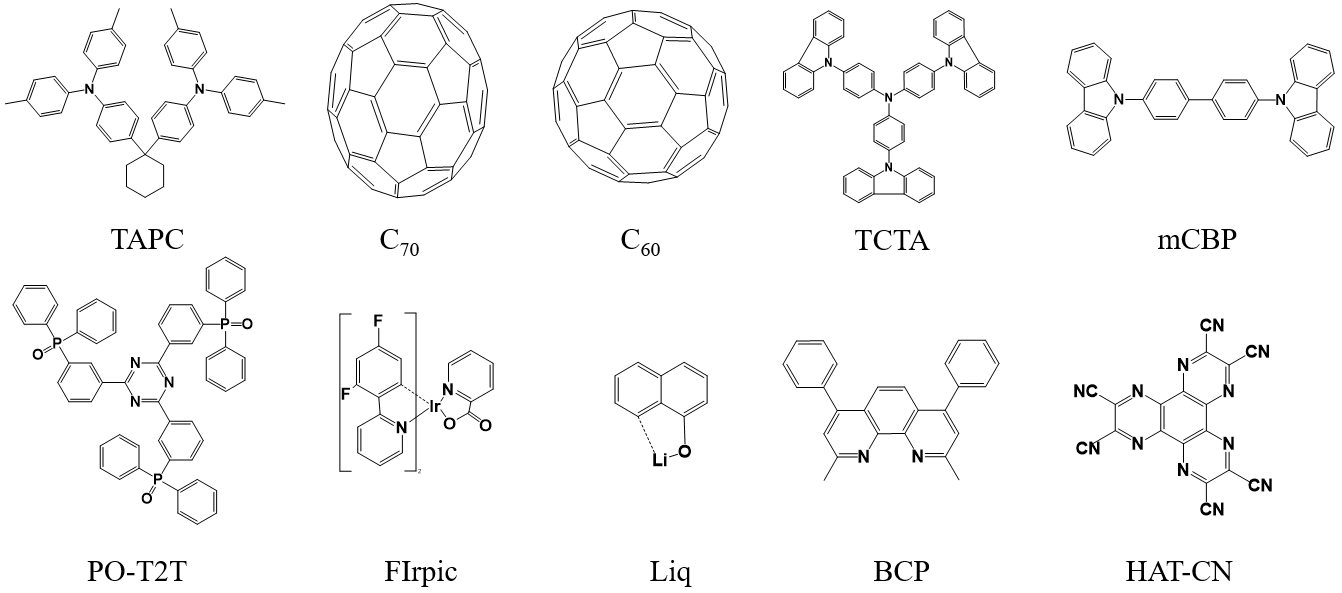


Figure S3. The chemical structure of the materials used.


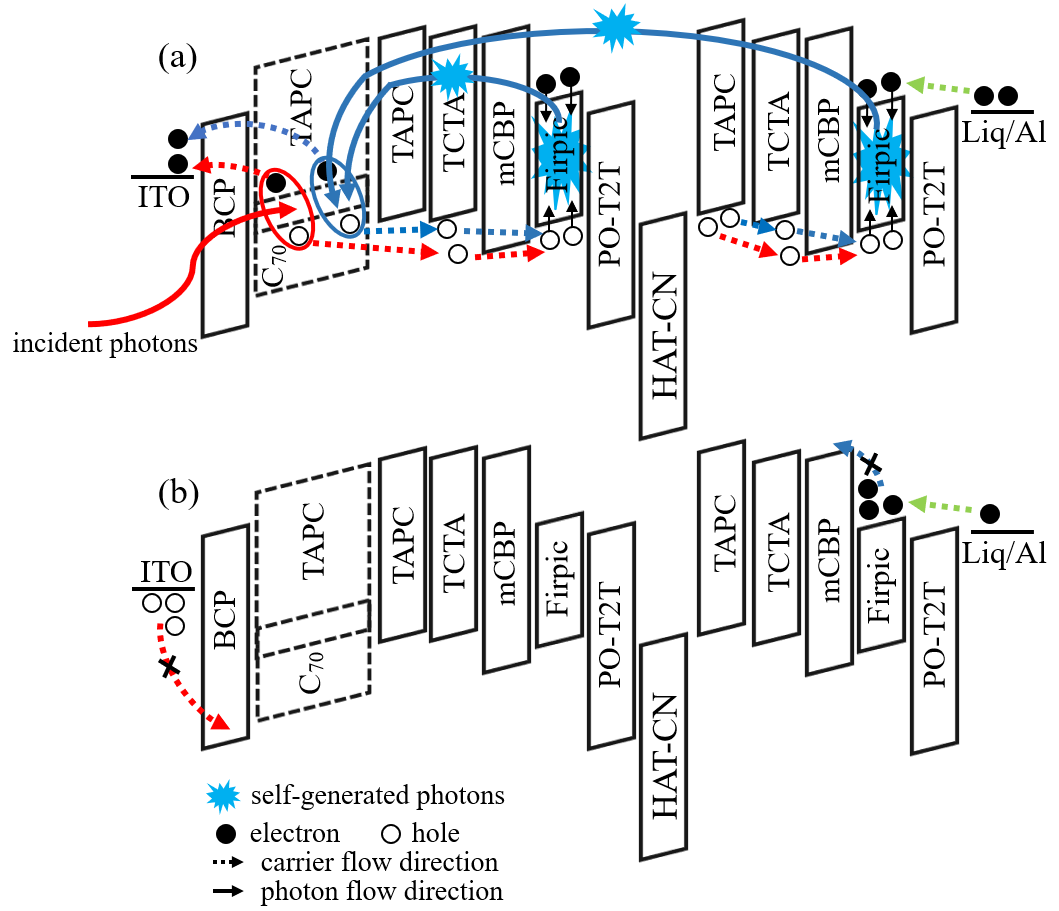


Figure S4. The working mechanism diagrams of the device a) under illumination and b) in the dark state, constructed based on the energy levels of the device.


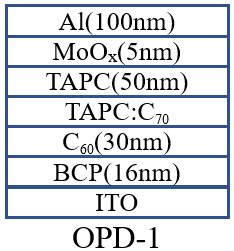


Figure S5. The detailed device structures of OPD-1.


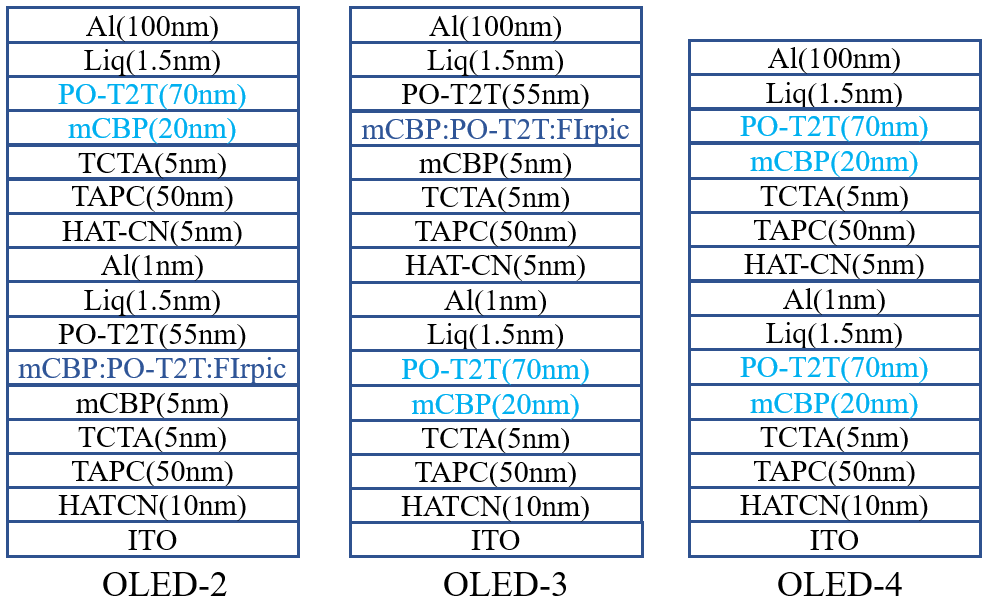


Figure S6. The detailed structures of OLED-2,3,4.

Figure S7. The EL spectra of OLED-1, 2,3,4.


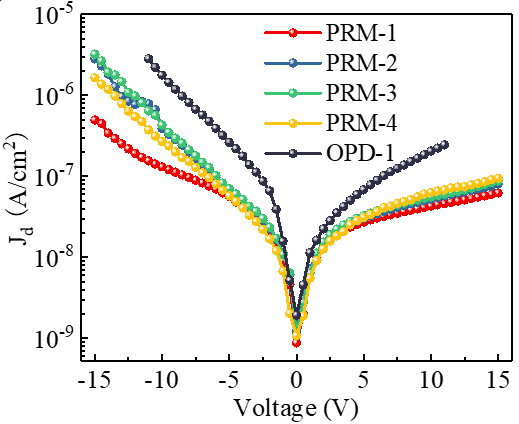


Figure S8. The dark current density-voltage curves of PRM-1, 2, 3, 4 and OPD-1.


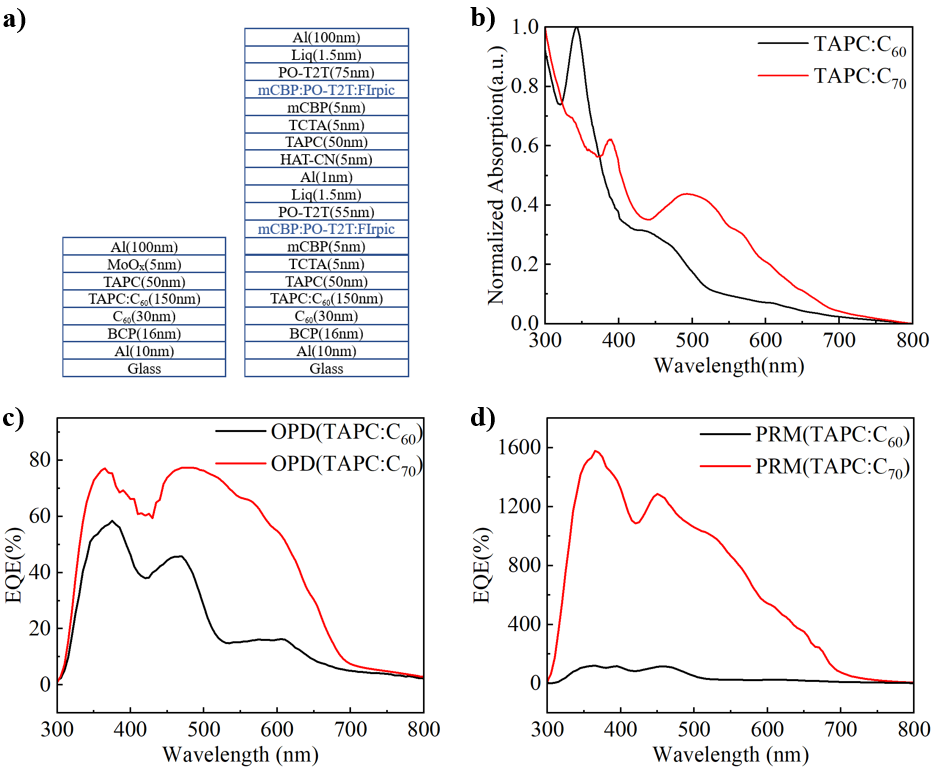


Figure S9. a) The detailed structures of OPD and PRM with TAPC:C_60_ blend films as the PSL. b) UV–vis absorption spectra of TAPC:C_60_ and TAPC:C_70_ blend films. *EQE* spectra of c) OPD and d) PRM with TAPC:C_60_ and TAPC:C_70_ blend films as the PSL, respectively.


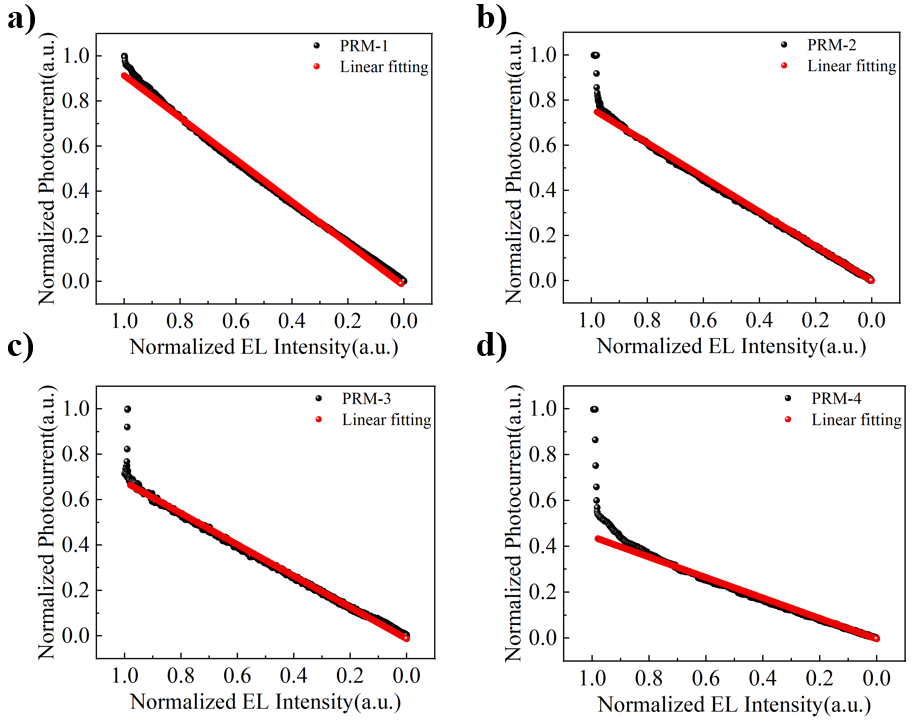


Figure S10. Characteristics and liner fitting curves of EL intensity-photocurrent for a) PRM-1, b) PRM-2, c) PRM-3, and d) PRM-4 in the descending phase, respectively.

Figure S11. The characteristics of *EQE –* *V* of OPD-1 under different wavelengths of light irradiation.


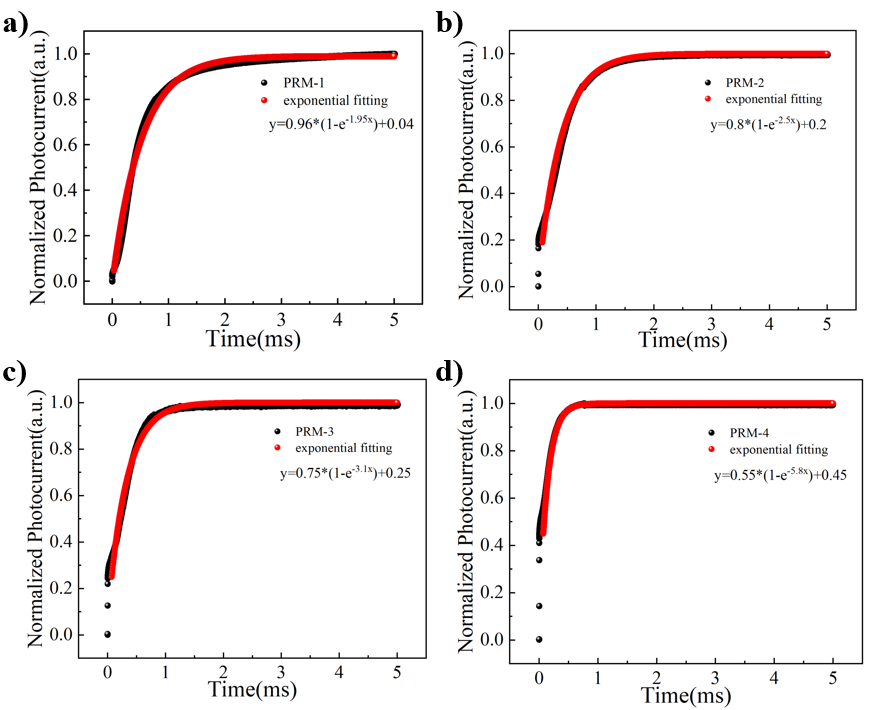


Figure S12. The transient photocurrent characteristics and exponential fitting curves of a) PRM-1, b) PRM-2, c) PRM-3, and d) PRM-4 in the ascending phase, respectively.


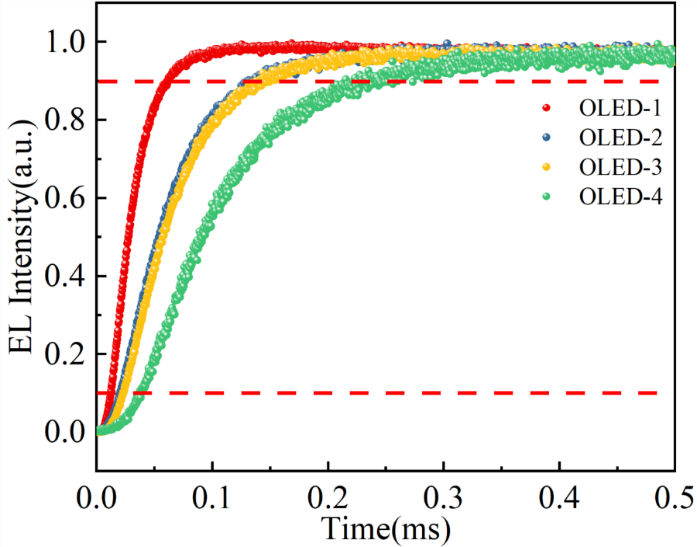


Figure S13. The transient photocurrent characteristics and exponential fitting curves of a) OLED-1, b) OLED-2, c) OLED-3, and d) OLED-4 in the ascending phase, respectively.


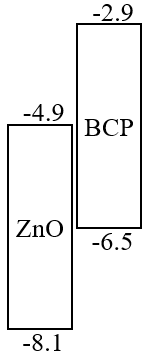


Figure S14. The energy level diagrams of BCP and ZnO.


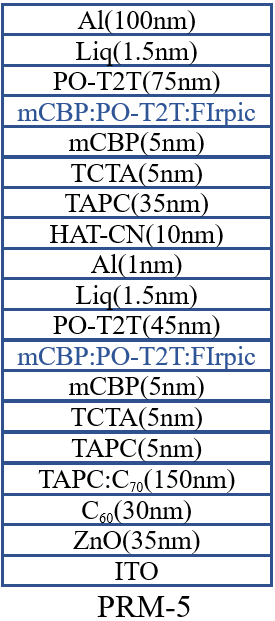


Figure S15. The detailed structure of PRM-5 and OPD-2 integrated device.


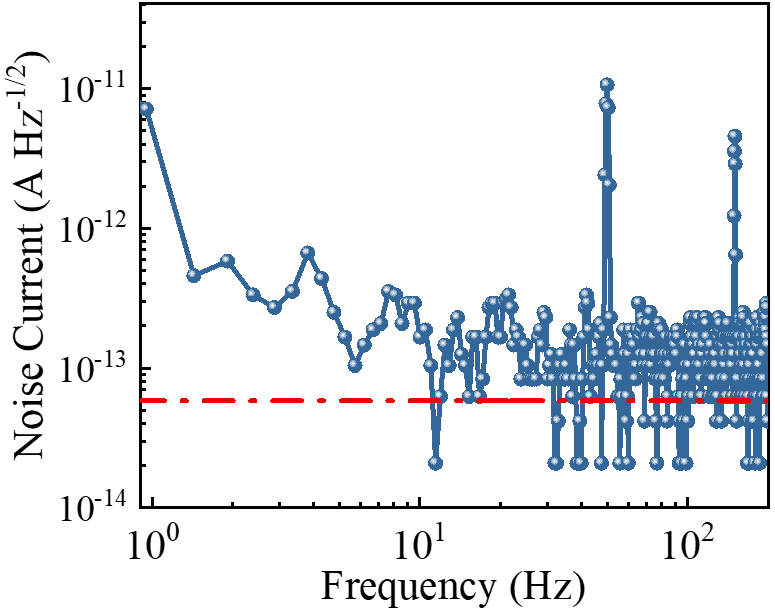


Figure S16. Measured noise spectra of the PRM-5 with a device area of 0.16 cm^2^ under 7.5V bias. The dashed line is the shot noise calculated from dark current.


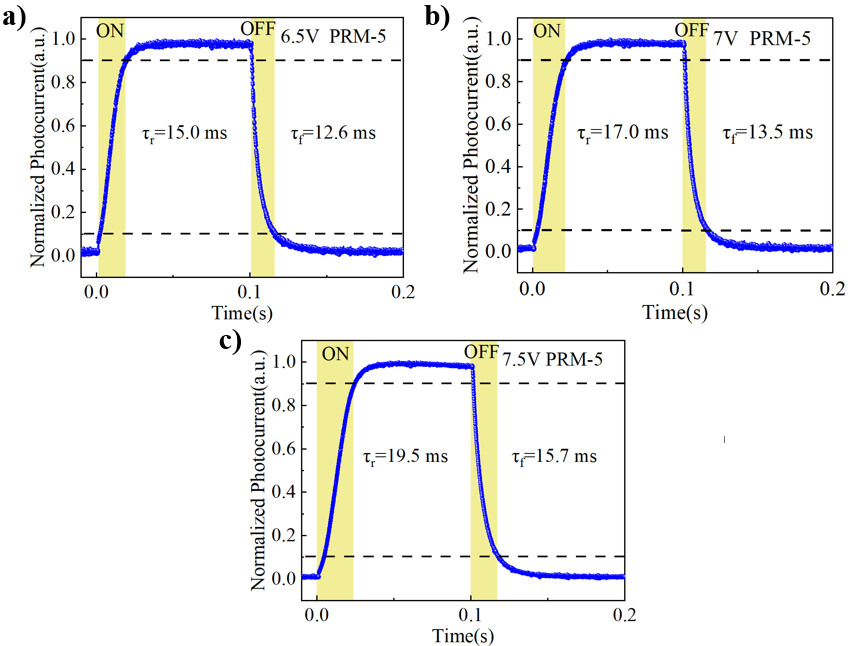


Figure S17. The transient photocurrent responses (*J–t* curves) of PRM-5 at a pulse frequency of 100 Hz with a device area of 0.2 cm^2^ measured by LED illumination with a peak of 520 nm under a) 6.5 V, b) 7 V and c) 7.5 V bias, respectively.


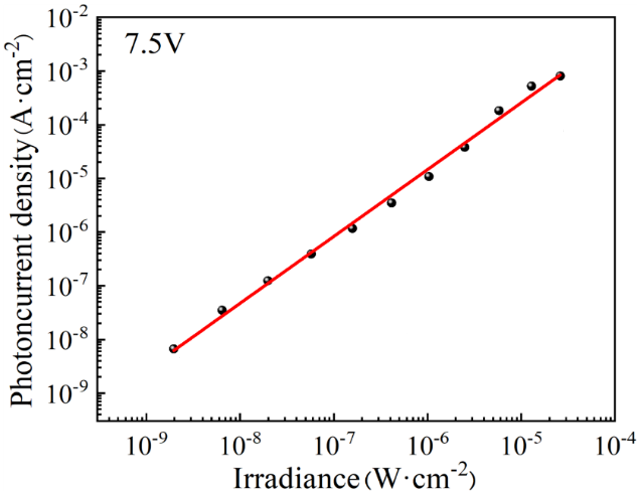


Figure S18. Incident light intensity-photocurrent density of PRM-5 under 7.5 V.


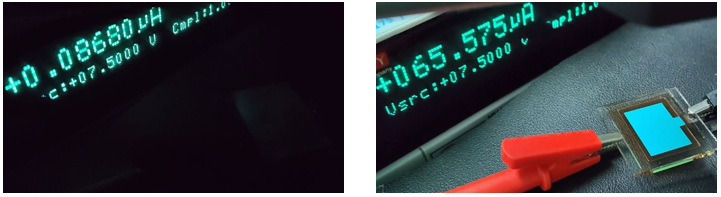


Figure S19. The schematic diagrams of large area PRM-OPD operation under dark state (left) and fluorescent illumination (right).


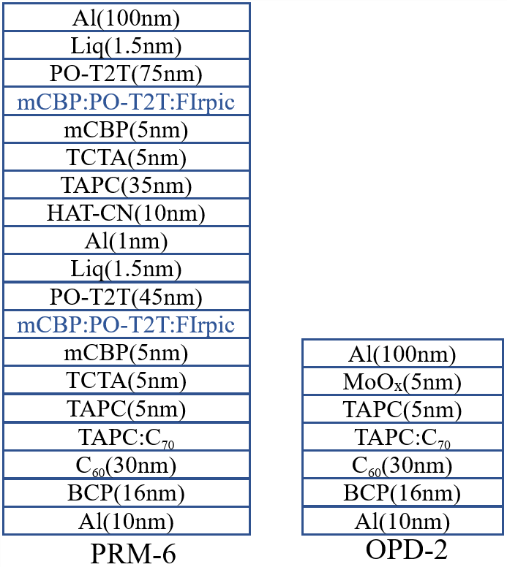


Figure S20. The detailed structures of PRM-6 and OPD-2.

Supplementary Notes

Supplementary Note 1: $\begin{matrix} \alpha_{1}\approx0.57 \\ \alpha_{2}\approx0.38 \end{matrix}$

For Equation 2:

$$1-\left(\alpha_{1}\eta_{EML1}\text{+}\alpha_{2}\eta_{EML2}\right)\eta_{PSL}\text{=}\frac{\eta_{A}\eta_{PSL}}{EQE}$$

The above formula represents the proportion of the photocurrent formed in T_0_ to the total photocurrent, which can be obtained from the inset in Figure 3a-d. Here, we define *η*_EML1_ and *η^’^*_EML1_as the high and low efficiency for different EML1, and define *η*_EML2_ and *η^’^*_EML2_ as the high and low efficiency for different EML2. In addition, considering that the OLED units in our devices have very similar spectra, it can be roughly assumed α_1_ and α_2_ that are only related to the distance between EML and PSL. Therefore, equations 2 of PRM-1,2,3,4 are as follows：

$0.96\text{=}\left(\alpha_{1}\eta_{EML1}\text{+}\alpha_{2}\eta_{EML2}\right)\eta_{PSL}$ (1)

$0.78\text{=}\left(\alpha_{1}\eta_{EML1}\text{+}\alpha_{2}\eta^{'}_{EML2}\right)\eta_{PSL}$ (2)

$0.7\text{=}\left(\alpha_{1}\eta^{'}_{EML1}\text{+}\alpha_{2}\eta_{EML2}\right)\eta_{PSL}$ (3)

$0.55\text{=}\left(\alpha_{1}\eta^{'}_{EML1}\text{+}\alpha_{2}\eta^{'}_{EML2}\right)\eta_{PSL}$ (4)

Considering that the tandem OLEDs in PRM-1 and PRM-4 have the same luminescent system, it can be assumed that *η_EML1_* is approximately equal to *η_EML2_*, and *η^’^_EML1_* is approximately equal to *η^’^_EML2_*.

$0.96\text{=}\left(\alpha_{1}\text{+}\alpha_{2}\right)\eta_{EML}\eta_{PSL}$ (5)

$0.55\text{=}\left(\alpha_{1}\text{+}\alpha_{2}\right)\eta^{'}_{EML}\eta_{PSL}$ (6)

From the expression (5)/(6), we can obtain:

$\eta^{'}_{EML}\text{=}\frac{0.55}{0.96}\eta_{EML}$ (7)

Substitute equation (7) into equations (2) and (3):

 (8)

$0.7\text{=}\left(\alpha_{1}\frac{0.55}{0.96}\eta_{EML}\text{+}\alpha_{2}\eta_{EML}\right)\eta_{PSL}$ (9)

From the expression (8)/(9), we can obtain:

$\alpha_{2}\approx0.67\alpha_{1}$ (10)

Substitute equation (10), as well as *η_EML_*≈1 and *η_PSL_*≈1 into equation (1):

$\begin{matrix} \alpha_{1}\approx0.57 \\ \alpha_{2}\approx0.38 \end{matrix}$ (11)

Supplementary Note 2: $T=\frac{\ln0.96}{-1.95}\approx21\mu s$

For PRM-1, the fitting formula is:

$y(x)=0.96(1-e^{-1.95x})+0.04$ (12)

For the normalized photocurrent：

$\frac{y(x)}{I_{In}}=\frac{0.96(1-e^{-1.95x})+0.04}{I_{In}}=EQE_{Nor}=\frac{EQE}{EQE_{\max}}$ (13)

*I_In_* indicates the incident photocurrent power. Establish a relation between formula 11 and formula 13 as:

$\begin{matrix} EQE\left(nT\right)=\eta_{A}\eta_{PSL}+\frac{\eta_{A}\eta_{PSL}\alpha\eta_{PSL}\eta_{EML}(1-\left[\alpha\eta_{PSL}\eta_{EML}\right]^{n-1})}{1-\alpha\eta_{PSL}\eta_{EML}} \\ =\frac{0.96(1-e^{-1.95x})+0.04}{I_{In}}EQE_{\max}=EQE \end{matrix}$ (14)

Equal-term relation:

$\begin{matrix} \eta_{A}\eta_{PSL}=EQE_{\max}\frac{0.04}{I_{In}} \\ \frac{\eta_{A}\eta_{PSL}\alpha\eta_{PSL}\eta_{EML}(1-\left[\alpha\eta_{PSL}\eta_{EML}\right]^{n-1})}{1-\alpha\eta_{PSL}\eta_{EML}}=\frac{0.96(1-e^{-1.95x})}{I_{In}}EQE_{\max} \end{matrix}$ (15)

After simplification:

$0.96^{n-1}=e^{-1.95x}$ (16)

T can be expressed as follows:

$T=\frac{x}{n-1}$ (17)

Take logarithms of both sides of the equation (15):

$\ln0.96=-1.95T$ (18)

$T=\frac{\ln0.96}{-1.95}\approx21\mu s$ (19)

For PRM-2, PRM-3, and PRM-4, using the same calculation method, their T can be obtained as 89 μs,93 μs,103 μs, respectively.

**Reference**

[1] Z. Zhao, J. Wang, C. Xu, K. Yang, F. Zhao, K. Wang, X. Zhang, F. Zhang, *J. Phys. Chem. Lett.* **2020**, 11, 2, 366–373

[2] M. Liu, J. Miao, J. Wang, Z. Zhao, K. Yang, X. Zhang, H. Peng, F. Zhang, *J. Mater. Chem. C*, **2020**, 8, 9854.

[3] Z. Zhao, B. Liu, C. Xu, M. Liu, K. Yang, X. Zhang, Y. Xu, J. Zhang, W. Li and F. Zhang, *J. Mater. Chem. C,* **2021**, 9, 5349.

[4] Z. Zhao, B. Liu, C. Xu, L. Li, M. Liu, K. Yang, S. Y. Jeong, H. Y. Woo, G. Yuan, W. Li and F. Zhang, *J. Mater. Chem. C*, **2022**,10, 7822-7830.

[5] S. Xing, J. Kublitski, C. Hänisch, L. C. Winkler, T.-y. Li, H. Kleemann, J. Benduhn, K. Leo, *Advanced Science*, **2022**, 9, 2105113.

[6] X. Shi, J. Qiao, S. Jeong, P. Lu, X. Du, H. Yin, W. Qin, H. Woo, X. Hao, *Appl. Phys. Lett.* 123, 233301 (**2023**).

[7] M. I. Kim, S. Lee, J. Kang, J. Kim, Z. Wu, J. H. Won, S. Baek, D. S. Chung, J. Y. Kim, I. H. Jung, H. Y. Woo, *Adv. Mater.* **2024**, 36, 2312396.

[8] J. Gao, Z. Wang, Y. Tang, J. Han, M. Gao, J. Wu, Q. Chen, D, Yu. E. Wang, F. Zhu, *ACS Appl. Mater. Interfaces* **2025**, 17, 37, 52426–52434.

[9] H. Qu, Z. Zhuo, X. Zhao, S. Zhang, X. Ma, Y. Zou, K. Yang, F. Zhang, *Adv. Optical Mater.* **2025**, 13, e01632.

[10] S. Zhang, L. Lu, C. Kang, H. Qu, X. Zhao, Y. Xie, X. Ma, Z. Zhuo, G. Qi, Q. Fan, K. Yang, and F. Zhang, *ACS Applied Materials & Interfaces* **2025,** 17, (27), 39375-39382.

[11] Y. Huang, L. Shao, Y. Wang, L. Hao, X. Luo, J. Zheng, Y. Cao, S. Li, Z. Tan, S. Li, W. Zhong, S. Dong, X. Yang, J. Benduhn, C. Liu, K. Leo, F. Huang, *Advanced Materials*, **2025**, 37, 2500491.
